# Optimization of embryogenic callus development in sugarcane suspension culture via the application of macronutrients and anti-browning agents

**DOI:** 10.1016/j.jgeb.2026.100853

**Published:** 2026-11-02

**Authors:** Parawita Dewanti, Agus Wedi Pratama, Nurmalasari Darsono, Purnama Okviandari, Tri Ratnasari, Rahmaniah Putri, Akbar Maulana

**Affiliations:** a Department of Agronomy, Faculty of Agriculture, University of Jember, Jember, Indonesia; b Center for Development of Advanced Science and Technology (CDAST), University of Jember, Jember, Indonesia; c Department of Agrotechnology, Faculty of Agriculture, University of Jember, Jember, Indonesia; d Research Center for Genetic Engineering, National Research and Innovation Agency (BRIN), Cibinong, Bogor, Indonesia

**Keywords:** Somatic embryogenesis, Sugarcane, Suspension culture, Embryogenic callus, Browning, Polyvinylpyrrolidone, Macronutrients

## Abstract

Liquid suspension culture can accelerate embryogenic callus proliferation in sugarcane, a polyploid crop that is difficult to breed conventionally. However, its efficiency is limited by nutrient composition and by phenolic oxidation, which causes browning and cell death. This study aimed to optimize embryogenic callus proliferation of sugarcane cv. Bulu Lawang in suspension culture by (i) supplementing Murashige and Skoog (MS) medium with potassium dihydrogen phosphate (KH_2_PO_4_; 0, 85, or 170 mg/L) and ammonium nitrate (NH_4_NO_3_; 0, 412, or 825 mg/L) in a factorial combination, and (ii) adding polyvinylpyrrolidone (PVP) or ascorbic acid (0–350 mg/L) as anti-browning agents. Callus induced from young spindle leaves was cultured in liquid medium for three weeks, and fresh weight, settled volume, morphology, cellular anatomy, and browning (culture color and cell viability) were assessed (ANOVA and Duncan's multiple range test, *p* < 0.05). Supplementation with 85 mg/L KH₂PO₄ and 825 mg/L NH₄NO₃ (final concentrations: 255 and 2475 mg/L) gave the highest fresh weight (0.53 g) and settled volume (12.0 cm^3^ at week 3, versus 5.64–7.84 cm^3^ in other combinations), with more embryogenic cells (small, with prominent nuclei). At 250 mg/L, both anti-browning agents significantly increased callus volume relative to controls (final volumes of 8.81 cm^3^ with PVP and 8.24 cm^3^ with ascorbic acid), reduced dead cells, shifted culture color from dominant brown to brownish white, and increased embryogenic and living cells. These findings define a nutrient and anti-browning regime that supports embryogenic callus proliferation in sugarcane suspension culture.

## Highlights

• Balanced KH_2_PO_4_ and NH_4_NO_3_ raised callus fresh weight 2.7-fold.
• The same nutrient mix increased settled callus volume by 57%.
• Phosphate alone did not improve growth; nitrogen balance was key.
• PVP or ascorbic acid at 250 mg/L reduced browning and dead cells.
• A narrow effective range supports embryogenic callus proliferation.

## Graphical abstract

**Figure:**
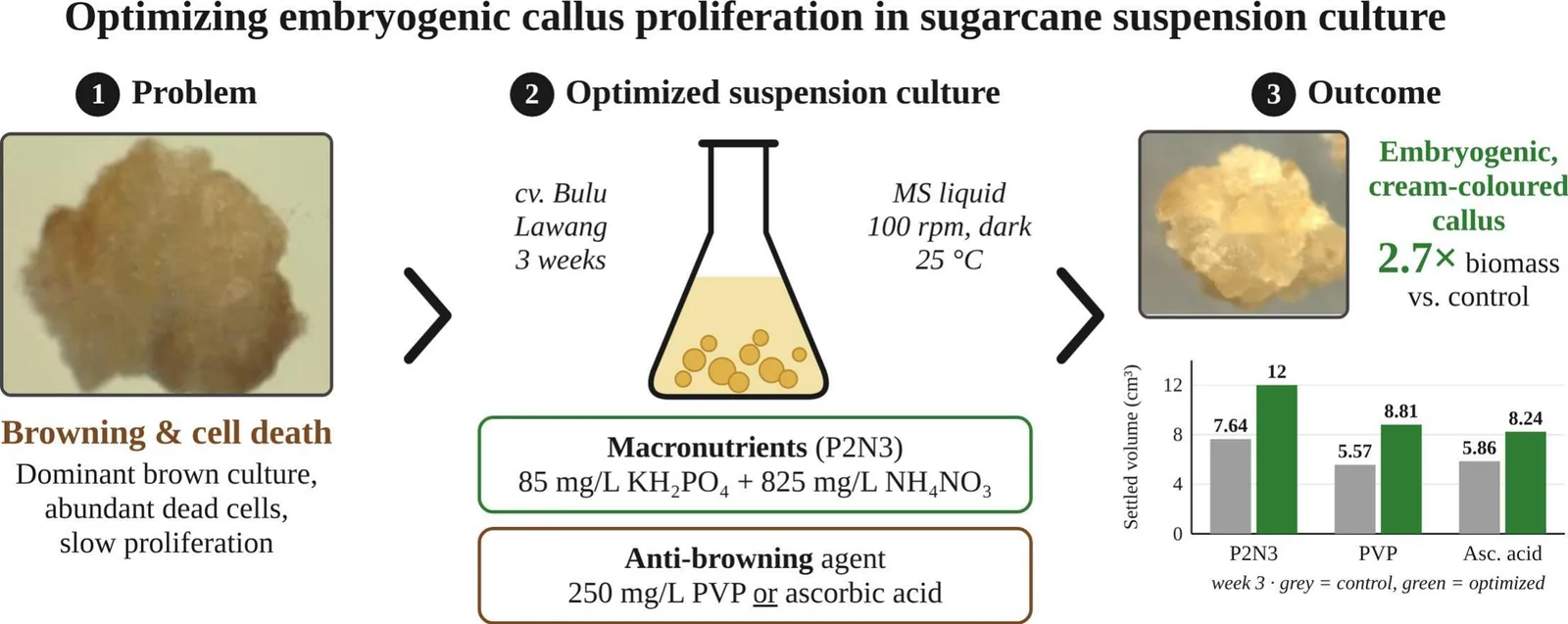

## 1 Introduction

Sugarcane (*Saccharum* spp. hybrids) is a major crop of tropical and subtropical regions, cultivated mainly for its sucrose content and increasingly as a renewable energy feedstock.^1^ Sugar, biofuels, animal feed, and industrial materials derived from it support extensive agro-industrial value chains.^2^, ^3^ Nevertheless, conventional sugarcane breeding is slow and labor-intensive because of the crop's complex polyploid genome, asynchronous flowering, and limited pollen viability.^4^ In vitro approaches therefore offer a practical means of bypassing these bottlenecks.^5^

Plant tissue culture is widely applied for clonal propagation, disease elimination, rejuvenation of aging cultivars, and the development of stress-tolerant clones.^6^ Shoots may be regenerated directly from meristematic tissues such as shoot tips and axillary buds (direct organogenesis) or indirectly through a callus phase. When somatic cells within the callus develop progressively into embryos and then plantlets, the pathway is termed indirect somatic embryogenesis,^7^ which depends on an adequate supply of high-quality embryogenic callus. In sugarcane, induction, proliferation, and embryo development have traditionally been carried out on solid media.^8^

Liquid suspension culture is a promising alternative. Continuous agitation disperses embryogenic aggregates uniformly, which can accelerate proliferation and embryo development and improve regenerative capacity.^9^, ^10^ Suspension systems also allow nutrient uptake and abiotic stress responses to be studied under relatively homogeneous conditions.^11^, ^12^ In sugarcane, however, few studies have attempted plant regeneration from suspension cultures, largely because of the technical difficulties involved.^13^

The performance of embryogenic cultures depends strongly on medium composition.^14^ Nitrogen is supplied mainly as ammonium (NH₄^+^) and nitrate (NO₃^−^), and both their ratio and total amount influence growth and morphogenesis in vitro.^15^ Phosphorus, supplied as KH₂PO₄, is required for cell division, protein synthesis, and callus development,^16^, ^17^ whereas potassium contributes to enzyme activation and the progression of embryogenesis.^18^ The optimal supply, however, is species- and stage-dependent. In cassava, for example, friable embryogenic callus formation was enhanced under reduced nitrate, potassium, and phosphate,^16^ and a recent sugarcane single-cell regeneration system used half-strength MS macronutrients at the regeneration stage.^19^ The nutrient regime that best supports embryogenic callus proliferation in liquid medium therefore cannot be inferred from other stages or species and must be determined empirically.

Browning is a second major constraint in vitro. Phenolic compounds released from wounded or stressed tissues are oxidized by endogenous enzymes into toxic quinones, which darken the medium, impair cell viability, and often lead to necrosis.^20^, ^21^, ^22^, ^23^, ^24^ Activated charcoal, ascorbic acid, and polyvinylpyrrolidone (PVP) are widely used to suppress browning, but their efficacy depends on the concentration applied, the species, and the culture system.^25^ PVP acts mainly as a phenolic adsorbent, whereas ascorbic acid is an antioxidant that scavenges reactive oxygen species and prevents quinone accumulation,^25^, ^26^ which makes a direct comparison of the two informative. In sugarcane, PVP has been reported to suppress browning during explant establishment and embryogenic callus initiation,^25^ but information on browning control during the proliferation of embryogenic cells in suspension remains limited.

Recent sugarcane studies have largely optimized callus induction, regeneration, and acclimatization protocols,^27^ or single-cell isolation and regeneration through hormonal regulation.^19^ These works address the induction and regeneration stages. The intermediate proliferation stage in liquid medium, which determines the quantity and quality of embryogenic material available for regeneration, has received comparatively little attention with respect to macronutrient supply and browning control. To our knowledge, the effects of KH₂PO₄ and NH₄NO₃ supplementation and of PVP versus ascorbic acid on biomass, embryogenic cell composition, and browning have not been systematically evaluated in sugarcane suspension culture.

Accordingly, this study aimed to optimize the proliferation of embryogenic callus of sugarcane cv. Bulu Lawang in suspension culture through two experiments: (i) supplementation of Murashige and Skoog (MS) medium with KH₂PO₄ and NH₄NO₃, and (ii) addition of PVP or ascorbic acid as anti-browning agents. Callus fresh weight, settled volume, cellular morphology, and browning were assessed to identify conditions that sustain embryogenic proliferation and can be integrated into sugarcane regeneration protocols.

## 2 Materials and methods

### 2.1 Plant material and explant preparation

Sugarcane (*Saccharum* spp.) cv. Bulu Lawang (BL) was obtained from the Sukosari Research Center, Lumajang, East Java, Indonesia. Young leaf whorls (spindle leaves) from 4- to 6-month-old plants were used as explants. To minimize contamination, the outer leaf sheaths were briefly dipped in 96% ethanol and flamed. Under aseptic conditions, the sheaths were peeled to a core diameter of 5 mm and sliced transversely into 2-mm-thick sections on sterile filter paper.

### 2.2 Callus induction and proliferation on solid medium

All media were based on Murashige and Skoog (MS) salts and vitamins (Table 1), adjusted to pH 5.8, solidified with 2.5 g/L Phytagel from Sigma, and autoclaved at 121 °C, 17.51 psi, for 30 min. Cultures were incubated in darkness at 25 °C. For callus induction, explants were cultured for six weeks on MS medium supplemented with 4 mg/L 2,4-dichlorophenoxyacetic acid (2,4-D), 300 mg/L casein hydrolysate, and 30 g/L sucrose, with subculturing every three weeks, following Dewanti et al.^28^ To promote embryogenic development, the induced calli were then transferred for three weeks to MS medium containing 2 mg/L 2,4-D, 500 mg/L casein hydrolysate, 560 mg/L L-proline, 40 mg/L l-glutamine, 50 mg/L L-arginine, and 30 g/L sucrose.

**Table 1:** Composition of Murashige and Skoog (MS) basal medium for callus induction and proliferation.

No	Compound	Amount (mg/L)
1	NH₄NO₃	1650
2	KNO₃	1900
3	KH₂PO₄	170
4	H₃BO₃	6.200
5	KI	0.830
6	Na₂MoO₄·2H₂O	0.250
7	CoCl₂·6H₂O	0.025
8	CaCl₂·2H₂O	440
9	MgSO₄·7H₂O	370
10	MnSO₄·4H₂O	22.300
11	ZnSO₄·4H₂O	8.600
12	CuSO₄·5H₂O	0.025
13	Na₂EDTA	37.300
14	FeSO₄·7H₂O	27.800
15	Vitamins:	
	Myo-inositol	100
	Pyridoxine-HCl	0.500
	Thiamine-HCl	0.100

### 2.3 Suspension culture and experimental treatments

Embryogenic calli from the solid proliferation stage were transferred to 100 mL flasks containing 25 mL of liquid basal medium (0.5 g callus per flask). The basal medium consisted of MS salts (4 g/L), 2 mg/L 2,4-D, 500 mg/L casein hydrolysate, 560 mg/L L-proline, 40 mg/L l-glutamine, 50 mg/L L-arginine, and 30 g/L sucrose at pH 5.8. Two experiments were conducted (Table 2).

**Table 2:** Composition of liquid proliferation media. (A) Macronutrient treatments (Experiment 1); final concentrations include the KH₂PO₄ and NH₄NO₃ already present in MS medium (170 and 1650 mg/L). (B) Anti-browning treatments (Experiment 2), added separately to the basal liquid medium.

Code	KH₂PO₄ added (mg/L)	NH₄NO₃ added (mg/L)	Final KH₂PO₄ (mg/L)	Final NH₄NO₃ (mg/L)
P1N1 (control)	0	0	170	1650
P1N2	0	412	170	2062
P1N3	0	825	170	2475
P2N1	85	0	255	1650
P2N2	85	412	255	2062
P2N3	85	825	255	2475
P3N1	170	0	340	1650
P3N2	170	412	340	2062
P3N3	170	825	340	2475


Anti-browning agent	Concentrations (mg/L)
PVP	0 (control), 200, 250, 300, 350
Ascorbic acid	0 (control), 200, 250, 300, 350

*Experiment 1 (macronutrients).* KH₂PO₄ (0, 85, or 170 mg/L) and NH₄NO₃ (0, 412, or 825 mg/L) were added to the basal medium in a 3 × 3 factorial combination, giving nine treatments (P1N1–P3N3). Because MS medium already contains 170 mg/L KH₂PO₄ and 1650 mg/L NH₄NO₃ (Table 1), the final concentrations ranged from 170 to 340 mg/L and from 1650 to 2475 mg/L, respectively. The unsupplemented basal medium (P1N1) served as the control.

*Experiment 2 (anti-browning agents).* PVP or ascorbic acid was added separately to the basal medium at 0 (control), 200, 250, 300, or 350 mg/L, without additional KH₂PO₄ or NH₄NO₃.

Flasks were placed on an orbital shaker at 100 rpm in darkness at 25 °C using an intermittent regime of 2 h shaking followed by 6 h rest, repeated continuously. After initial dispersion (24 h), the speed was reduced to 80 rpm to help separate callus aggregates. Cultures were maintained for three weeks with subculturing at weekly intervals. Subculturing was performed by removing the callus inoculum, weighing it using an analytical balance, and transferring it to fresh medium with the same composition and treatment as the previous culture.

### 2.4 Data collection

#### 2.4.1 Callus fresh weight and settled volume

Callus fresh weight (*W*_c_) was determined by differential weighing. The flask containing 25 mL of liquid medium was weighed before inoculation (*W*₁). After three weeks, the callus aggregates were harvested and finely sectioned, and the flask containing the medium and callus was weighed (*W*₂):

(1) $$W_{c}=W_{2}-W_{1}$$

Settled callus volume (*V*_c_) was measured weekly. The suspension was allowed to settle for 15 min, and the height of the callus sediment (*h*_p_, mm) and the total height of the liquid including the sediment (*h*_t_, mm) were measured:

(2) $$V_{c}=\left(h_{p}/h_{t}\right)\times V_{m}$$

where *V*_m_ is the initial medium volume (25 cm^3^).

#### 2.4.2 Callus morphology and cellular anatomy

Callus structure and somatic embryo stages (globular, scutellar, and coleoptilar) were observed under a stereo microscope (Olympus CX23). Cellular anatomy was examined at 400× magnification under an Olympus CX23 after 1 mL of the sample was placed on the microscope slide using a micropipette. Embryogenic cells were identified by their small size and prominent nuclei, and non-embryogenic cells by their elongated shape.

#### 2.4.3 Cell composition, viability, and browning

The abundance of embryogenic, non-embryogenic, living, and dead cells was scored semi-quantitatively (+, sparse; ++, moderate; +++, abundant), and the color of the suspension was recorded visually as an indicator of browning (Table 4). Cell viability (*C*_*v*_) was assessed every two weeks by collecting a 10 μL cell suspension sample from each treatment under sterile conditions and mixing it with 10 μL of trypan blue solution in a microtube. The mixture was loaded onto a hemocytometer and observed under a light microscope at 100× magnification. The number of living cells (*C*_*l*_) and dead cells (*C*_*d*_) was counted in the 4 × 4 grid. Cell viability (*C*_*v*_) was calculated as follows:

(3) $$C_{v}\;\left(\%\right)=\left(C_{l}/C_{t}\right)\times 100$$

where *C*_*t*_ represents the total number of cells, calculated as the sum of living cells (*C*_*l*_) and dead cells (*C*_*d*_).

#### 2.4.4 Statistical analysis

Experiment 1 was arranged as a 3 × 3 factorial in a completely randomized design, and Experiment 2 as a completely randomized design, each with 3 replicate flasks per treatment conducted independently. Quantitative data were subjected to analysis of variance (ANOVA), and means were compared using Duncan's multiple range test (DMRT) at *p* < 0.05 in IBM SPSS Statistics, version 25. Semi-quantitative data were analyzed descriptively.

## 3 Results

### 3.1 Development of embryogenic callus on solid medium

On induction medium containing 4 mg/L 2,4-D, explants began to swell, mainly near the cut edges, between weeks 2 and 4 (Fig. 1). By week 6, two callus types could be distinguished (Fig. 2). Embryogenic callus was yellowish-white, friable, and nodular, and contained pre-embryogenic masses (PEM) and globular embryogenic cells (GE) (Fig. 2b). Non-embryogenic callus was compact and brown (Fig. 2a). Some explants showed progressive browning that ended in blackening and necrosis. After transfer to proliferation medium (2 mg/L 2,4-D), embryogenic callus expanded and appeared translucent and glossy, whereas non-embryogenic callus enlarged but remained dense, moist, and brownish-yellow (Fig. 3). From week 8, most embryogenic callus was at the globular stage, and structures marking the onset of the scutellar stage were visible (Fig. 3b and c).

**Fig. 1:**
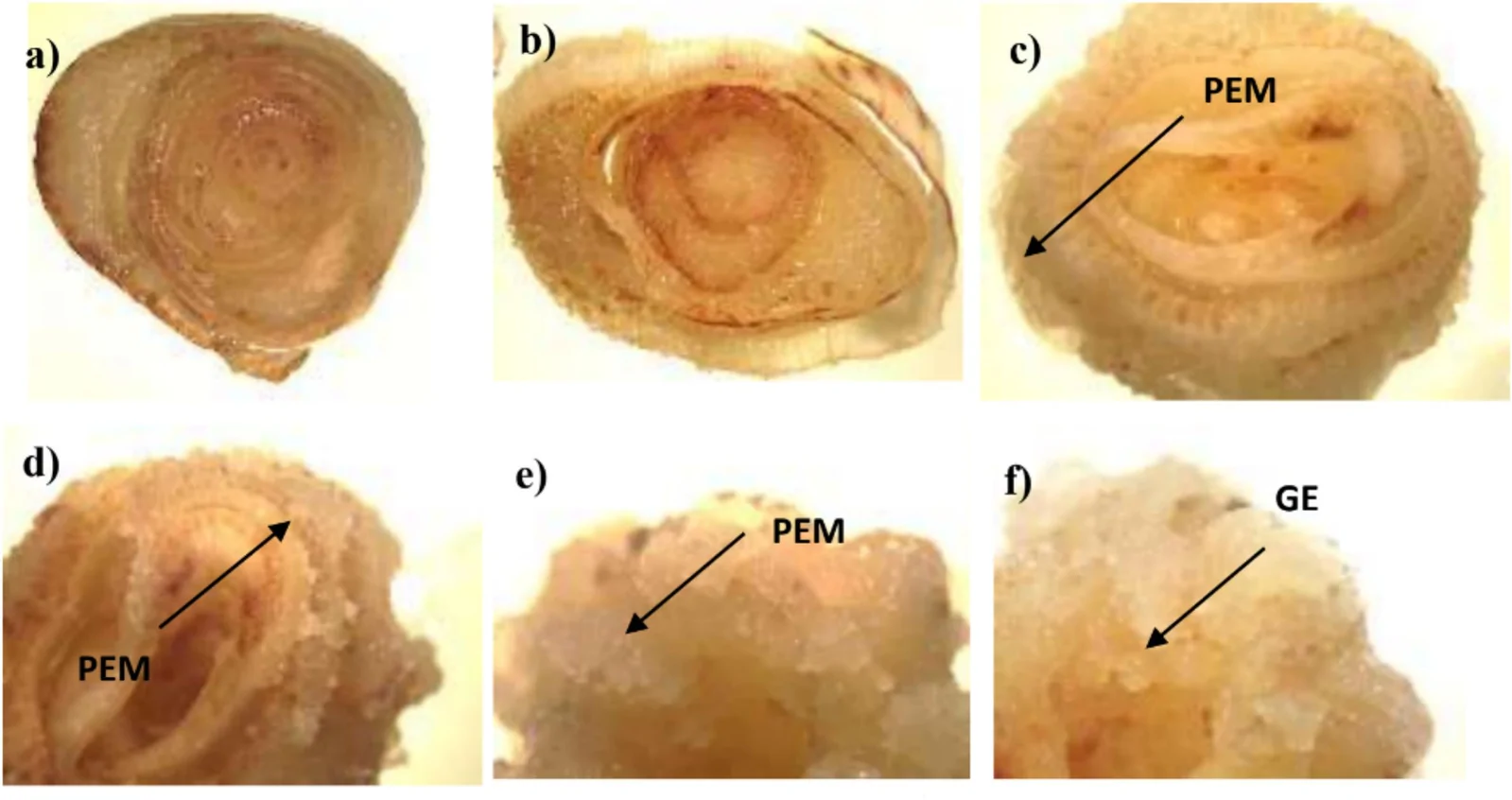
Morphological development of sugarcane spindle leaf explants during the callus induction stage from week 1 to week 6 (a–f). Scale bar = 1 mm. Abbreviations: GE, globular embryogenic cells; PEM, pre-embryogenic mass.

**Fig. 2:**
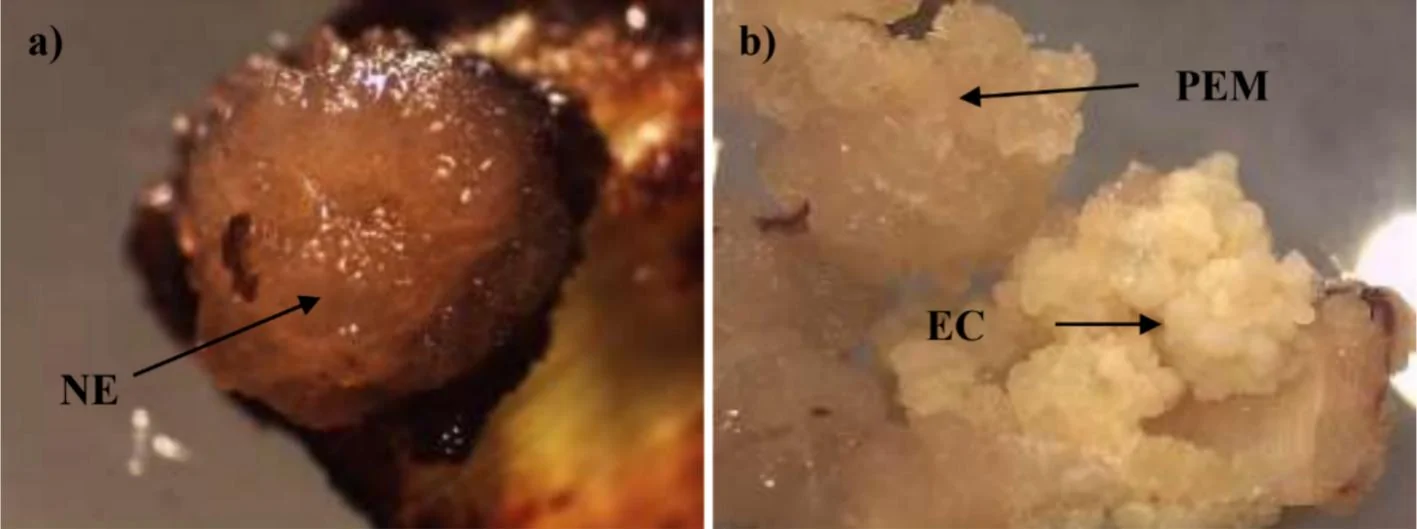
Morphological comparison of (a) non-embryogenic and (b) embryogenic callus at six weeks of culture. Abbreviations: NE, non-embryogenic cells; PEM, pre-embryogenic mass; EC, embryogenic cells.

**Fig. 3:**
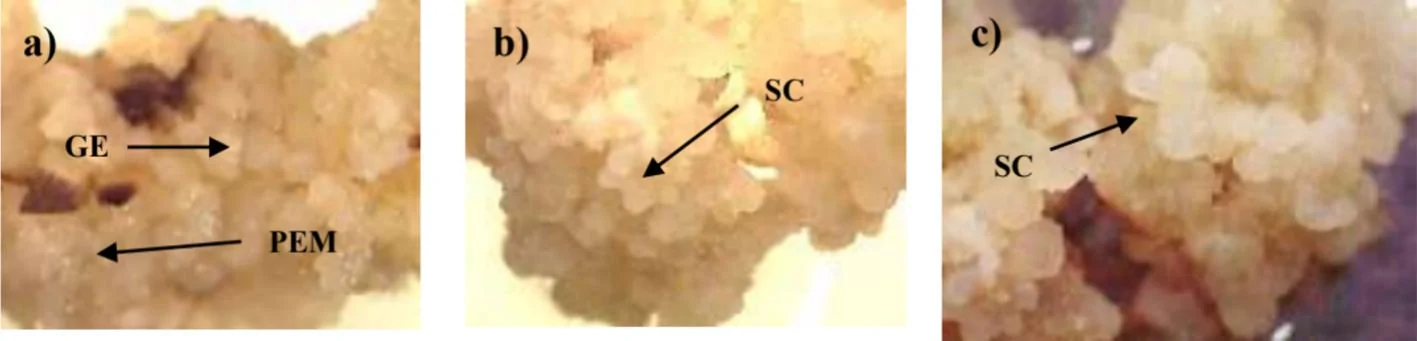
Morphological progression of embryogenic callus on solid proliferation media at (a) 7 weeks, (b) 8 weeks, and (c) 9 weeks. Abbreviations: GE, globular embryogenic cells; PEM, pre-embryogenic mass; SC, scutellum cells.

### 3.2 Effect of macronutrient supplementation on callus proliferation in suspension culture

Macronutrient supplementation significantly affected callus fresh weight and settled volume (Table 3). ANOVA showed that KH₂PO₄ and NH₄NO₃ had highly significant effects on callus fresh weight and settled volume, and their interaction was significant (*p* < 0.01). After three weeks, treatment P2N3 (85 mg/L KH₂PO₄ and 825 mg/L NH₄NO₃ added; final concentrations 255 and 2475 mg/L) gave the highest fresh weight (0.53 g), 2.7 times that of the unsupplemented control P1N1 (0.20 g). The lowest fresh weight (0.16 g) occurred in P2N1, and all other treatments ranged from 0.19 to 0.31 g. P2N3 also had the largest settled volume at every time point, significantly greater than all other treatments, increasing from 6.39 cm^3^ at week 1 to 9.55 cm^3^ at week 2 and 12.0 cm^3^ at week 3. At week 3, the volume in P2N3 was 57% larger than in the control (7.64 cm^3^), while the other treatments ranged from 5.64 to 7.84 cm^3^.

**Table 3:** Effect of macronutrient combinations (KH₂PO₄ and NH₄NO₃ added to MS medium) on the settled volume and fresh weight of embryogenic callus in suspension culture. Values are means of 3 replicates; means followed by the same letter within a column are not significantly different according to DMRT (*p* < 0.01).

Media composition	Settled callus volume (cm^3^)			Fresh weight (g)
	Week 1	Week 2	Week 3	
P1N1 (0 mg/L KH₂PO₄ + 0 mg/L NH₄NO₃)	4.41 cd	5.64 bc	7.64 b	0.20 bc
P1N2 (0 mg/L KH₂PO₄ + 412 mg/L NH₄NO₃)	5.05 bc	5.56 bc	6.30 cd	0.22 bc
P1N3 (0 mg/L KH₂PO₄ + 825 mg/L NH₄NO₃)	5.62 b	6.35 b	7.44 bc	0.31 b
P2N1 (85 mg/L KH₂PO₄ + 0 mg/L NH₄NO₃)	4.24 d	4.79 c	5.64 d	0.16 c
P2N2 (85 mg/L KH₂PO₄ + 412 mg/L NH₄NO₃)	5.48 b	6.25 b	6.99 bc	0.30 b
P2N3 (85 mg/L KH₂PO₄ + 825 mg/L NH₄NO₃)	6.39 a	9.55 a	12.0 a	0.53 a
P3N1 (170 mg/L KH₂PO₄ + 0 mg/L NH₄NO₃)	4.29 d	5.34 bc	6.97 bc	0.19 c
P3N2 (170 mg/L KH₂PO₄ + 412 mg/L NH₄NO₃)	5.37 bc	6.41 b	7.84 b	0.26 bc
P3N3 (170 mg/L KH₂PO₄ + 825 mg/L NH₄NO₃)	4.99 cd	5.62 bc	6.88 bc	0.24 bc

The response depended on the combination of salts. Adding KH₂PO₄ alone did not improve growth (0.16–0.19 g in P2N1 and P3N1 versus 0.20 g in the control). At 85 mg/L KH₂PO₄, fresh weight rose with NH₄NO₃ (0.16, 0.30, and 0.53 g at 0, 412, and 825 mg/L). Raising KH₂PO₄ to 170 mg/L with 825 mg/L NH₄NO₃ (P3N3) did not further increase growth (0.24 g; 6.88 cm^3^).

Microscopic observation showed small embryogenic cells (EC) with prominent nuclei and elongated non-embryogenic cells (NE) in the suspensions (Fig. 4). P2N3 contained a higher proportion of embryogenic cells than P1N1 as the control. Fresh weight in the P2N3 treatment increased by 112%, whereas in the P1N1 control, fresh weight decreased by 20% (calculated based on the initial inoculum weight of 0.25 g) (Table 3).

**Fig. 4:**
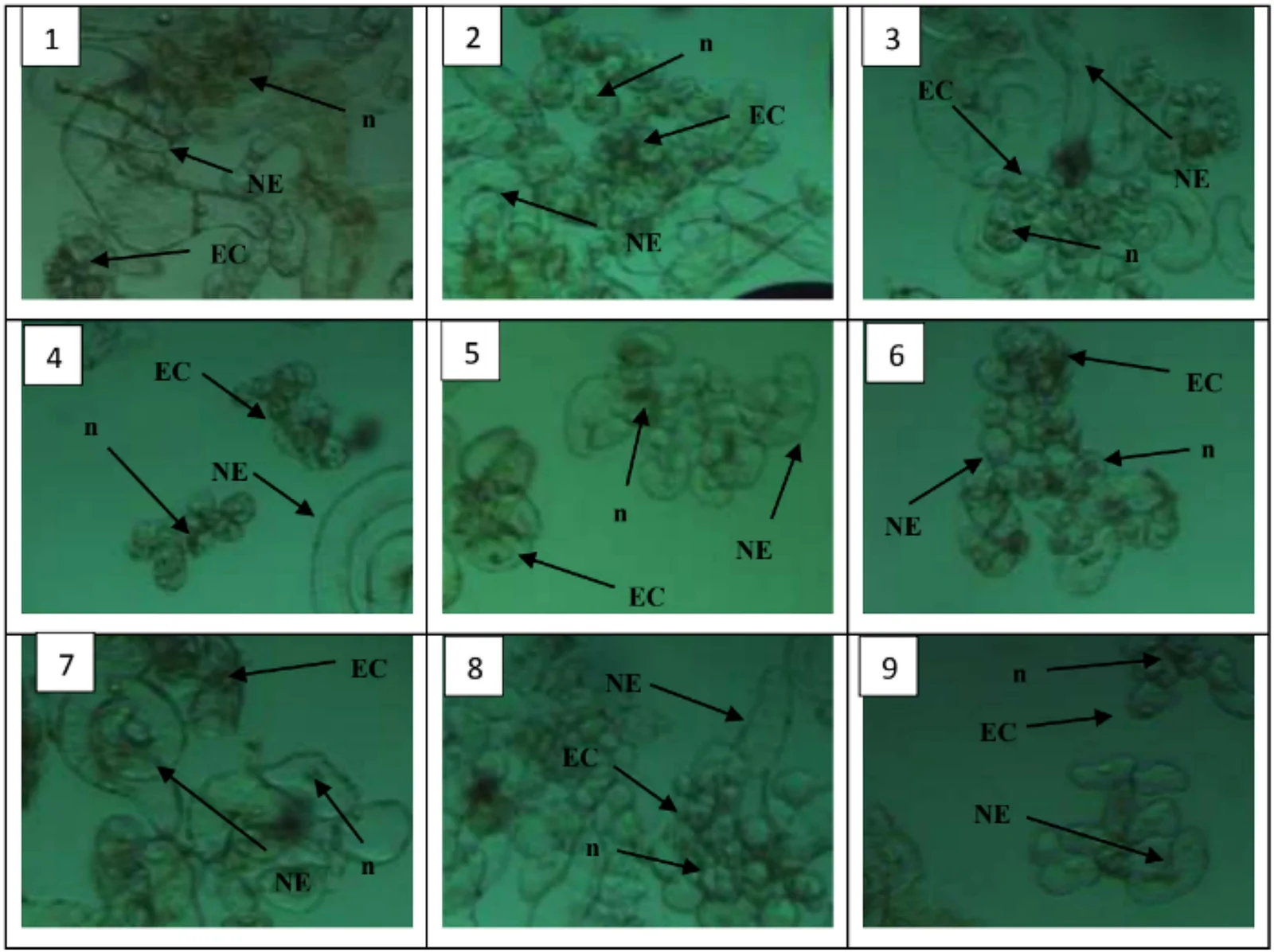
Microscopic observation (100× magnification) of cellular anatomy under varying macronutrient treatments: (1) P1N1 to (9) P3N3. Abbreviations: EC, embryogenic cells; NE, non-embryogenic cells; n, nucleus.

### 3.3 Effect of anti-browning agents on callus growth, cell condition, and browning

Both agents improved culture condition, with the best response at 250 mg/L (Table 4). Untreated suspensions were dominantly brown and contained abundant dead cells (+++) but few embryogenic (+) and living (+) cells. At 250 mg/L, both PVP and ascorbic acid gave abundant embryogenic and living cells (+++), few dead cells (+), and a brownish-white suspension. PVP at 300 mg/L also gave abundant embryogenic cells (+++) but fewer living cells (++). At 350 mg/L, suspensions were slightly brown and contained more dead cells (++ with PVP; +++ with ascorbic acid, as in the control) and fewer embryogenic cells (++ and +, respectively).

**Table 4:** Qualitative assessment of callus development in suspension culture treated with the anti-browning agents PVP and ascorbic acid. (+ = sparse; ++ = moderate; +++ = abundant).

Anti-browning agent	Concentration (mg/L)	Embryogenic cells	Non-embryogenic cells	Living cells	Dead cells	Color of suspension culture
PVP	0	+	++	+	+++	dominant brown
	200	++	+	++	+	slightly brown
	250	+++	+	+++	+	brownish white
	300	+++	+	++	+	brownish white
	350	++	+	+	++	slightly brown
Ascorbic acid	0	+	++	+	+++	dominant brown
	200	++	++	++	+	brownish white
	250	+++	+	+++	+	brownish white
	300	++	++	++	+	slightly brown
	350	+	++	+	+++	slightly brown

At 250 mg/L, both agents gave the largest settled volume at each week (Fig. 5), reaching 8.81 cm^3^ with PVP and 8.24 cm^3^ with ascorbic acid at week 3, significantly larger than their respective controls (at week 3 with results of 5.57 and 5.86 cm^3^). PVP at concentrations of 300 and 350 mg/L resulted in lower settled volumes than the control, and the settled volume also increased over the cultivation period with the changes over time being statistically evaluated. At 250 mg/L, the two agents produced similar qualitative responses (Table 4). The difference in final settled volume between PVP and ascorbic acid was statistically significant (*P* < 0.01).

**Fig. 5:**
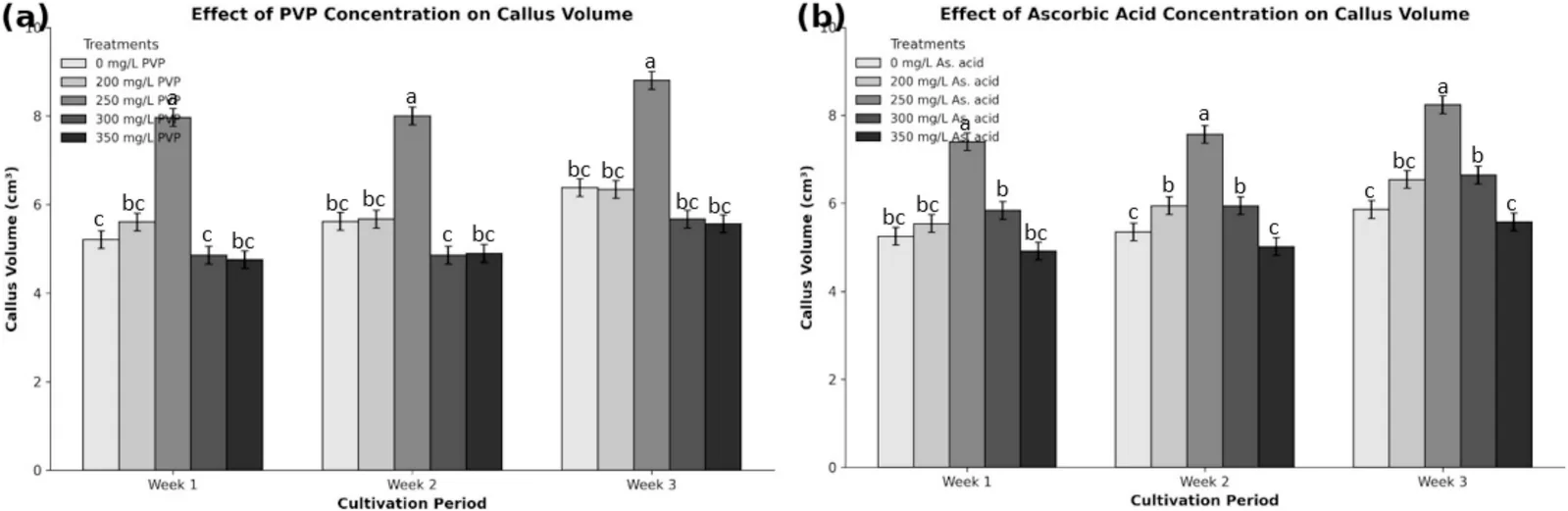
Effect of (a) PVP and (b) ascorbic acid concentrations on callus volume in liquid suspension culture over a three-week period. Means followed by the same letter within a column are not significantly different according to DMRT (*p* < 0.01).

After three weeks, aggregates from untreated suspensions were dark brown (Figs. 6e and 7e), whereas those treated with PVP or ascorbic acid were cream-colored to light brown, with some brown patches at 350 mg/L ascorbic acid (Fig. 7d). Globular embryogenic cells (GE) were observed at all concentrations, and scutellar structures (SC) at 250 mg/L PVP (Fig. 6b) and at 200 and 250 mg/L ascorbic acid (Fig. 7a and b). Cellular anatomy showed distinct embryogenic cells in suspensions treated with 250 mg/L PVP or ascorbic acid (round cells containing a nucleus) (Fig. 8c and g), whereas fewer embryogenic cells were observed in the control compared to non-embryogenic cells, which were characterized by an elongated shape (Fig. 8a).

**Fig. 6:**
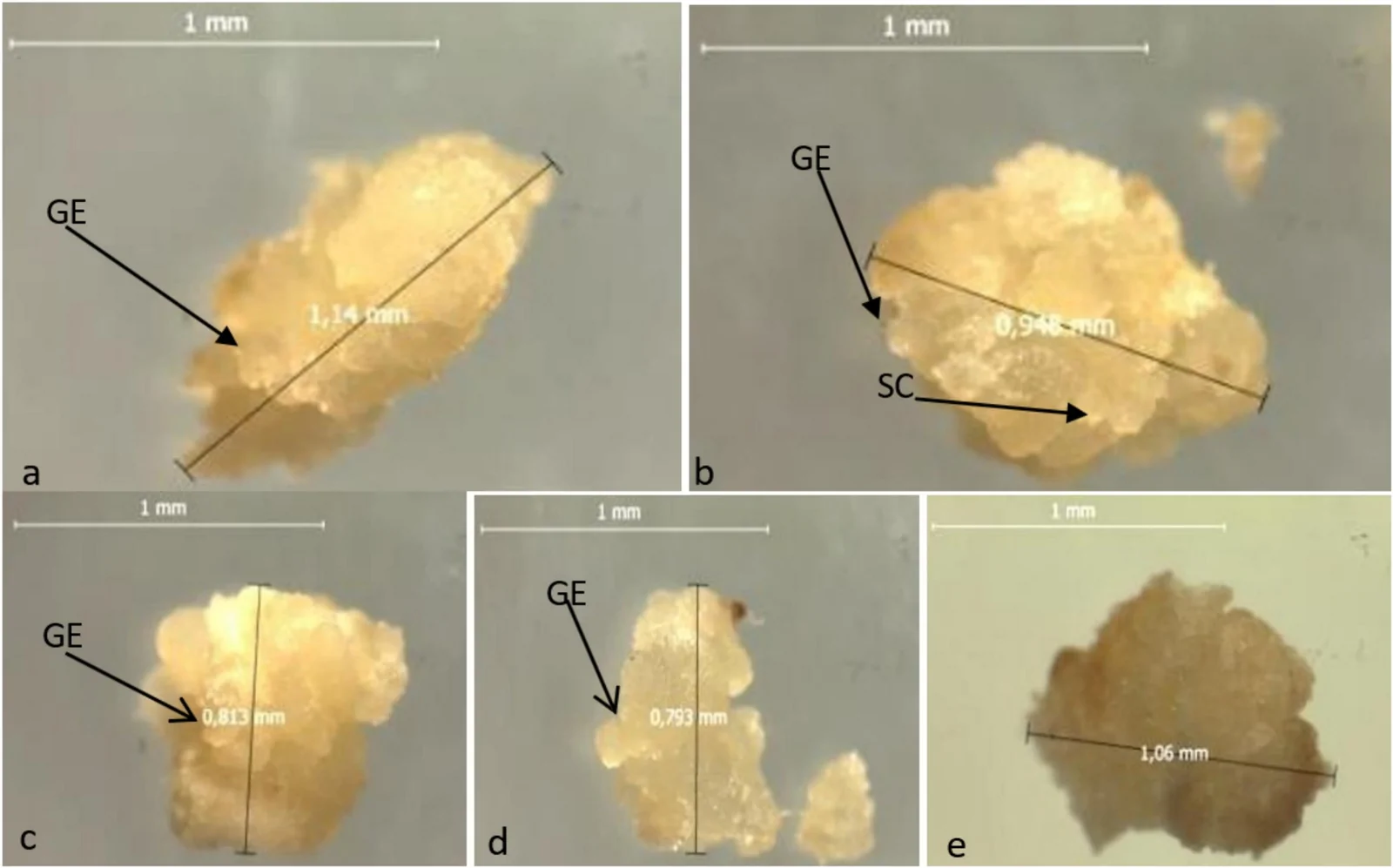
Callus morphology after three weeks of cultivation in liquid proliferation media supplemented with PVP: (a) 200 mg/L, (b) 250 mg/L, (c) 300 mg/L, (d) 350 mg/L, and (e) control. Abbreviations: GE, globular embryogenic cells; SC, scutellum cells.

**Fig. 7:**
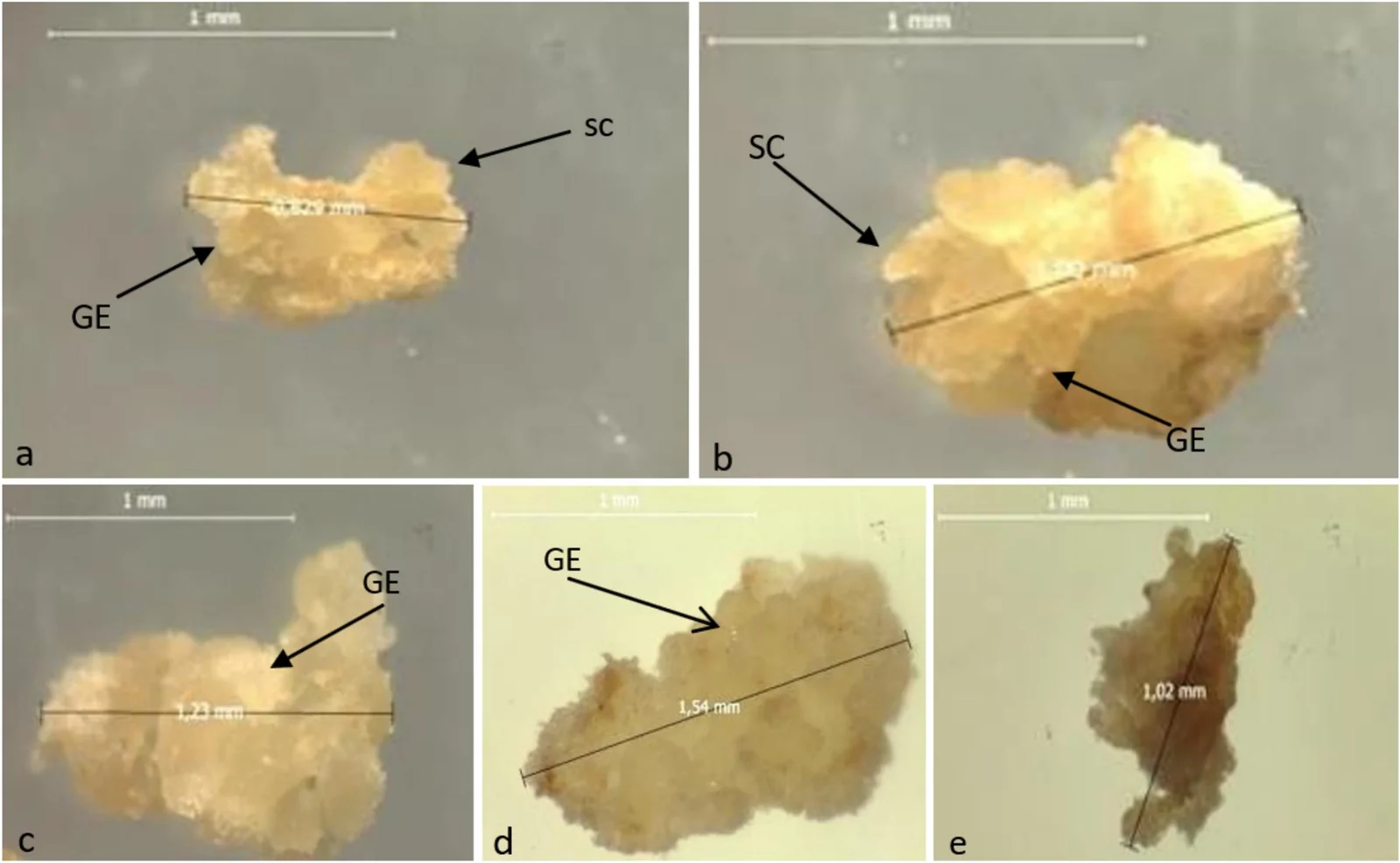
Callus morphology after three weeks of cultivation in liquid proliferation media supplemented with ascorbic acid: (a) 200 mg/L, (b) 250 mg/L, (c) 300 mg/L, (d) 350 mg/L, and (e) control. Abbreviations: GE, globular embryogenic cells; SC, scutellum cells.

**Fig. 8:**
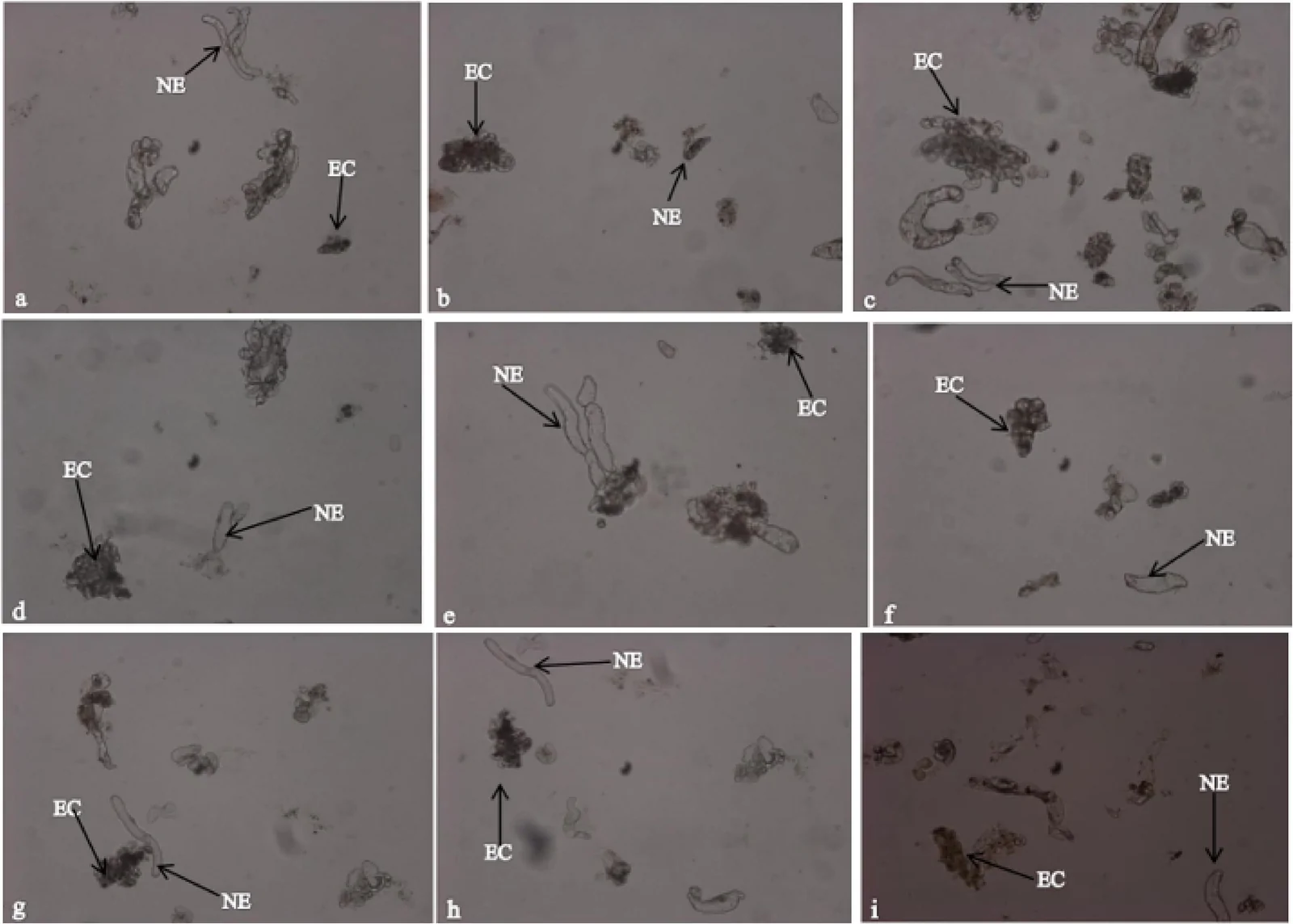
Microscopic observation (100× magnification) of cellular anatomy after three weeks in liquid proliferation media supplemented with anti-browning agents: (a) control; (b–e) PVP at 200–350 mg/L; and (f–i) ascorbic acid at 200–350 mg/L.

## 4 Discussion

### 4.1 Callus development on solid medium

The first phase of somatic embryogenesis is the dedifferentiation of explant tissue into callus, which begins with continuous cell division and appears as tissue swelling and clumping. The swelling near the cut edges between weeks 2 and 4 agrees with the observations of Busaifi and Hirjani.^29^ Induction with 4 mg/L 2,4-D produced embryogenic callus within six weeks,^28^ allowing the transition to pre-embryogenic masses (PEM) and then to globular stages. Reducing 2,4-D to 2 mg/L in the proliferation medium is consistent with the physiological shift from rapid cell division during induction to structural maturation, in which PEM develop into globular and scutellar stages. Similar timelines have been reported for other sugarcane cultivars, indicating that week 8 is a pivotal point for globular-stage maturation.^7^, ^30^ Transfer to liquid medium with intermittent agitation was intended to disperse callus aggregates and accelerate embryo development,^30^ and the aggregates were fragmented into a finely divided suspension whose settled volume increased over the three-week culture.

### 4.2 Nutrient balance governs embryogenic callus proliferation in suspension

The response pattern in Table 3 indicates that proliferation was governed by the balance between nitrogen and phosphate rather than by either nutrient alone. Adding KH₂PO₄ without additional NH₄NO₃ did not increase biomass, and raising KH₂PO₄ from 85 to 170 mg/L gave no further gain. In contrast, combining 85 mg/L KH₂PO₄ with 825 mg/L NH₄NO₃ raised fresh weight 2.7-fold and settled volume by 57% relative to the control. This suggests that nitrogen was the first limiting nutrient in the basal medium under our conditions and that phosphate became beneficial only once nitrogen was sufficient. Ammonium nitrate supplies both reduced (NH₄^+^) and oxidized (NO₃^−^) nitrogen, whose ratio and total amount influence growth and morphogenesis in vitro,^15^ and nitrogen availability strongly reshapes the metabolism of sugarcane suspension cells.^12^ Phosphate supports cell division and callus development.^16^, ^17^ The absence of further improvement at 340 mg/L final KH₂PO₄ (P3N3) may reflect ionic or osmotic stress or phosphate precipitation with medium cations, but these possibilities were not tested.

The benefit of higher macronutrient supply contrasts with reports for other systems and stages. Friable embryogenic callus formation in cassava was enhanced under reduced nitrate, potassium, and phosphate,^16^ and a recent sugarcane single-cell regeneration system used half-strength MS macronutrients at the regeneration stage.^19^ Together, these findings suggest that nutrient demand differs between the proliferation stage, when biomass accumulation is the priority, and the induction or differentiation stages, when a lower nutrient status may favor embryogenic commitment. Our data address only the proliferation stage, and whether the P2N3 regime should be reduced before regeneration remains to be tested.

### 4.3 Cellular composition of the suspensions

The higher biomass in P2N3 coincided with a higher proportion of small embryogenic cells with prominent nuclei relative to elongated non-embryogenic cells (Fig. 4), a cytological distinction also used to characterize cell suspension cultures.^13^, ^31^ This supports the interpretation that the added nutrients favored proliferation of the embryogenic fraction rather than merely expanding non-embryogenic cells. However, fresh weight and settled volume also reflect cell expansion and aggregate size, and our cell assessment was qualitative. Cell counts and the percentage of embryogenic cells are needed to confirm this interpretation.

### 4.4 Anti-browning agents are effective within a narrow concentration range

Browning arises from the oxidation of phenolic compounds released by stressed cells, and in suspension culture these compounds diffuse rapidly and darken the whole medium.^32^ The two agents act on this process differently: PVP adsorbs phenolics through hydrogen bonding and may inhibit enzymes involved in quinone formation,^25^, ^33^ whereas ascorbic acid is an antioxidant that scavenges reactive oxygen species and inhibits the oxidation cascade.^25^, ^26^ At 250 mg/L, both agents shifted the cultures from dominant brown to brownish white, reduced dead cells, and increased living and embryogenic cells (Table 4; Fig. 6, Fig. 7, Fig. 8). Scutellar structures were observed at 250 mg/L PVP and at 200–250 mg/L ascorbic acid (Figs. 6b, 7a and b). Reduced oxidative damage probably allowed more cells to remain viable and advance along embryogenic development, although phenolic content and polyphenol oxidase (PPO) activity were not measured. In peony, PVP at 0.5 g/L suppressed browning, and lower browning coincided with lower activities of phenylalanine ammonia-lyase and PPO,^34^ which are readouts that could confirm this mechanism in sugarcane.

The effect of the two agents on cell viability varied with concentration. The highest proportion of dead cells was observed in the PVP control (0 mg/L), whereas for ascorbic acid, the highest proportion of dead cells was observed at 0 and 350 mg/L (Table 4). Excess PVP may bind beneficial medium components such as growth regulators and nutrients, and excess ascorbic acid may become pro-oxidant or acidify the medium; neither mechanism was tested here. A similarly narrow optimum was reported for ascorbic acid in Norway spruce somatic embryogenesis, where 25 and 200 mg/L raised hydrogen peroxide levels and lowered endogenous hormone levels, whereas 50 mg/L gave the highest embryogenic tissue induction.^35^ Ascorbic acid has also been reported to work best in combination with citric acid,^36^ which suggests that combinations of agents deserve testing. Anti-browning agents should therefore be titrated for each species and culture system.

At 250 mg/L, PVP and ascorbic acid gave similar qualitative improvements (Table 4). The final volume for 250 mg/L PVP (8.81 cm^3^) was higher than control (5.57 cm^3^), and the final volume for 250 mg/L ascorbic acid (8.24 cm^3^) was higher than that of the control (5.86) (Fig. 5).

### 4.5 Implications and limitations

Earlier sugarcane studies have largely optimized induction and regeneration protocols.^19^, ^27^ The present results add quantitative information for the intermediate proliferation stage in liquid medium. Both macronutrient supplementation and browning control have narrow effective ranges, and within them they improved biomass, cell composition, and culture condition in cv. Bulu Lawang. P2N3 and 250 mg/L PVP or ascorbic acid are therefore candidate components of suspension media for sugarcane regeneration protocols.

Several limitations should be noted. First, only one cultivar was tested, and the cultures were not taken through regeneration, so the effect on plantlet recovery is unknown. Second, the anti-browning agents were tested in basal medium without P2N3 supplementation, so additive or interacting effects remain untested. Third, growth was assessed by fresh weight and settled volume, and cell status by semi-quantitative scoring; cell counts, packed cell volume, dry weight, and viability assays would provide stronger evidence. Fourth, biochemical markers of browning (phenolics, PPO activity, H₂O₂) were not measured. Finally, plants regenerated from suspension-derived material should be checked for genetic fidelity, as was done for sugarcane regenerated from cell suspension culture using RAPD and ISSR markers.^13^ Future work should combine P2N3 with PVP or ascorbic acid, extend the test to other cultivars, and follow the cultures through regeneration and acclimatization.

## 5 Conclusion

In suspension cultures of sugarcane cv. Bulu Lawang, supplementing MS medium with 85 mg/L KH₂PO₄ and 825 mg/L NH₄NO₃ (final concentrations 255 and 2475 mg/L) gave the highest callus fresh weight (0.53 g) and settled volume (12.0 cm^3^ after three weeks), together with a qualitatively higher proportion of embryogenic cells. Phosphate alone did not increase growth, and the highest phosphate level gave no further gain, indicating that nutrient balance rather than either salt alone determined the response. PVP or ascorbic acid at 250 mg/L reduced browning and dead cells, increased living and embryogenic cells and callus volume, and coincided with more advanced embryogenic structures, whereas 350 mg/L was less effective. The two agents were similarly effective at 250 mg/L in the qualitative assessments. These conditions define candidate components of suspension media for sugarcane regeneration protocols. Their effect on plantlet regeneration, their combined use, their performance in other cultivars, and the biochemical basis of the anti-browning response remain to be tested.

## CRediT authorship contribution statement

**Parawita Dewanti:** Writing – review & editing, Writing – original draft, Validation, Supervision, Formal analysis, Conceptualization. **Agus Wedi Pratama:** Writing – review & editing, Visualization, Supervision, Investigation. **Nurmalasari Darsono:** Writing – original draft, Visualization, Methodology, Investigation, Formal analysis. **Purnama Okviandari:** Resources, Investigation, Formal analysis, Data curation. **Tri Ratnasari:** Supervision, Resources, Investigation, Formal analysis. **Rahmaniah Putri:** Visualization, Resources, Methodology, Formal analysis. **Akbar Maulana:** Visualization, Resources, Methodology, Formal analysis.

## Ethics approval

Not applicable to this study.

## Declaration of competing interest

The authors declare that they have no known competing financial interests or personal relationships that could have appeared to influence the work reported in this paper.

## Data Availability

Data presented in this study will be available on a fair request to the corresponding author.
